# Exploring polypharmacy with artificial intelligence: data analysis protocol

**DOI:** 10.1186/s12911-021-01583-x

**Published:** 2021-07-20

**Authors:** Caroline Sirois, Richard Khoury, Audrey Durand, Pierre-Luc Deziel, Olga Bukhtiyarova, Yohann Chiu, Denis Talbot, Alexandre Bureau, Philippe Després, Christian Gagné, François Laviolette, Anne-Marie Savard, Jacques Corbeil, Thierry Badard, Sonia Jean, Marc Simard

**Affiliations:** 1 Faculty of Pharmacy, Université Laval, Quebec, QC Canada; 2 Quebec National Institute of Public Health, Quebec, QC Canada; 3 Faculty of Science and Engineering, Department of Computer Science and Software Engineering, Université Laval, Quebec, QC Canada; 4 Faculty of Law, Université Laval, Quebec, QC Canada; 5 Faculty of Medicine, Department of Social and Preventive Medicine, Université Laval, Quebec, QC Canada; 6 Faculty of Science and Engineering, Department of Physics, Physical Engineering and Optics, Université Laval, Quebec, QC Canada; 7 Faculty of Science and Engineering, Department of Electrical and Computer Engineering, Université Laval, Quebec, QC Canada; 8 Faculty of Medicine, Department of Molecular Medicine, Université Laval, Quebec, QC Canada; 9 Faculty of Forestry, Geography and Geomatics, Department of Geomatic Science, Université Laval, Quebec, QC Canada; 10 Centre d’excellence sur le vieillissement de Québec, Hôpital St-Sacrement, Local L2-28, 1050, chemin Ste-Foy, Quebec, QC G1S 4L8 Canada

**Keywords:** Polypharmacy, Indicators, Medications, Artificial intelligence, Ethics, Social acceptability

## Abstract

**Background:**

Polypharmacy is common among older adults and it represents a public health concern, due to the negative health impacts potentially associated with the use of several medications. However, the large number of medication combinations and sequences of use makes it complicated for traditional statistical methods to predict which therapy is genuinely associated with health outcomes. The project aims to use artificial intelligence (AI) to determine the quality of polypharmacy among older adults with chronic diseases in the province of Québec, Canada.

**Methods:**

We will use data from the Quebec Integrated Chronic Disease Surveillance System (QICDSS). QICDSS contains information about prescribed medications in older adults in Quebec collected over 20 years. It also includes diagnostic codes and procedures, and sociodemographic data linked through a unique identification number for each individual. Our research will be structured around three interconnected research axes: AI, Health, and Law&Ethics. The AI research axis will develop algorithms for finding frequent patterns of medication use that correlate with health events, considering data locality and temporality (explainable AI or XAI). The Health research axis will translate these patterns into polypharmacy indicators relevant to public health surveillance and clinicians. The Law&Ethics axis will assess the social acceptability of the algorithms developed using AI tools and the indicators developed by the Heath axis and will ensure that the developed indicators neither discriminate against any population group nor increase the disparities already present in the use of medications.

**Discussion:**

The multi-disciplinary research team consists of specialists in AI, health data, statistics, pharmacy, public health, law, and ethics, which will allow investigation of polypharmacy from different points of view and will contribute to a deeper understanding of the clinical, social, and ethical issues surrounding polypharmacy and its surveillance, as well as the use of AI for health record data. The project results will be disseminated to the scientific community, healthcare professionals, and public health decision-makers in peer-reviewed publications, scientific meetings, and reports. The diffusion of the results will ensure the confidentiality of individual data.

## Background

Medications represent a large proportion of health care spendings [1–7]. Their use is essential for older adults who suffer from multiple chronic diseases. Canadians aged 65 years and over receive on average 6.9 different classes of medications annually [8]. Polypharmacy, which is the simultaneous use of multiple medications by the same individual, has been associated with a plethora of harmful health consequences, such as frailty, falls, cognitive problems, hospitalizations, and mortality [9–11]. It thus represents a potential harm for the patient and a financial burden for the health care system [7, 12, 13].

Nonetheless, there are circumstances in which the prescription of multiple medications is appropriate and leads to improved health outcomes. Distinguishing appropriate and inappropriate polypharmacy is an important and complex issue that is difficult to tackle [14]. First, there is a plurality of definitions of polypharmacy [15, 16]. Some of them are based solely on quantitative aspects (for example, more than 4, 5 or 10 simultaneously used medications), while others are based on qualitative characteristics (for example, the presence of inappropriate medications), and some use mixed approaches [15, 16]. Second, each combination of different medications has a different risk/benefit ratio due to the variety of potential drug-drug and drug-disease interactions [17]. The individual characteristics and clinical manifestations are also important drivers of the polypharmacy type and consequences [18–20]. Finally, the concept of polypharmacy is often studied as a static exposure to medications without considering past medication use or subsequent changes, which may limit the conclusions about the consequences of polypharmacy [17].

Identifying what combinations of medications or trajectories of treatment are associated with health outcomes would allow the development of specific polypharmacy indicators for public health surveillance [21, 22]. Such indicators would be useful to guide clinical practice, to implement interventions or policies and would allow for their evaluation thereafter.

Determining health outcomes associated with polypharmacy requires considering concurrent and sequential use of multiple medications, duration of treatment, medical and sociodemographic characteristics of individuals. This type of analysis of large amount of complex data is difficult to perform with traditional statistics, but it can be accomplished with artificial intelligence (AI) methods.

However, using AI in large health administrative data may pose ethical challenges, such as those related to re-identifying individuals or using the data sparingly, that is to restrict attention only to the variables necessary to answer the research question. Moreover, the polypharmacy indicators that are created must neither marginalize nor discriminate against any specific population. Taking into consideration the ethical and social aspects involved will be beneficial for researchers, clinicians, decision-makers, and for the population in general.

### Aim

The general objective of the research program is to use AI within an ethical framework to develop polypharmacy indicators in older adults for surveillance and clinical practice in the province of Quebec, Canada.

**Fig. 1:**
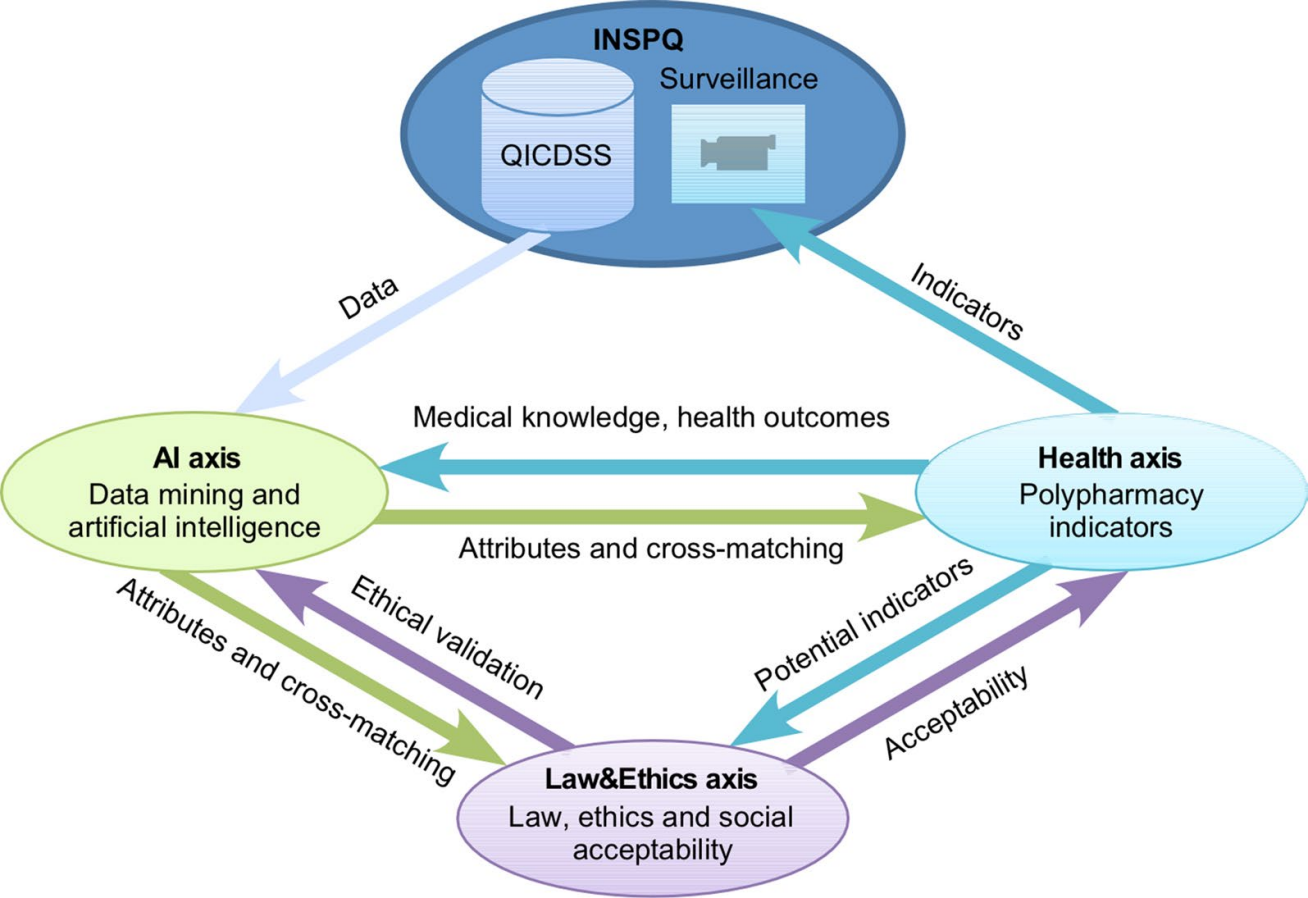
Collaboration between three research axes and the main knowledge user. Three research axes will be interrelated throughout the project. While the AI axis will start the analysis of the surveillance data of the QICDSS, the Health axis will carry out a literature review on health outcomes that could be fed into subsequent AI axis research. The first results of the AI axis will be transmitted to the other two axes, which will ensure their ethical justifiability, social acceptability (Law&Ethics axis) and clinical relevance and the ability to be translated into concrete indicators for surveillance (Health axis). Feedback from the Health and Law&Ethics axes to the AI axis will allow to refine or to reorient its research. The indicators developed by the Health axis will also be transferred to the Law&Ethics axis to verify their social acceptability. The INSPQ will be involved in all stages, and the polypharmacy indicators selected at the end of the project will be integrated into the chronic disease surveillance

Three interdependent research axes (Fig. 1) will be involved to reach this aim:

- AI axis: Define and detect polypharmacy in administrative databases to identify frequent combinations that correlate with health outcomes.
- Health axis: Develop polypharmacy indicators in older adults to carry out polypharmacy surveillance in public health and to guide clinicians.
- Law&Ethics axis: Explore the ethical and social acceptability aspects related to the use of AI for the development of polypharmacy indicators.

## Methods/design

### Data source

We will use the data from the Quebec Integrated Chronic Disease Surveillance System (QICDSS). This database was developed and is managed by the Quebec National Institute of Public Health (Institut national de santé publique du Québec, INSPQ) for the surveillance of chronic diseases [23]. It contains information about medication claims (name of prescribed medication, dose, dates of dispensation, duration of treatment), physician claims (dates, diagnostic codes based on the 9th version of the International Statistical Classification of Diseases and Related Health Problems [ICD-9]), hospitalizations (dates, diagnostic codes based on ICD-9 or ICD-10, provided services and interventions), deaths (date and up to 10 causes), and sociodemographic data (age, sex, region of residence). A unique identification number allows linking information from each file. The database also includes the material and social deprivation index, that is a validated substitute of socio-economic status [24], and a comorbidity index derived from Charlson and Elixhauser indices [25] that allows to quantify the burden of diseases of an individual. The QICDSS medication data cover more than 90% of the older population (65 years and older) since 1996.

### Methodological approach

The three research axes described herein will closely collaborate to achieve the objectives of the project. As shown in Fig. 1, the results generated by each of the axes will serve as input for the other two axes and will thus make it possible to adjust their work.

### AI axis: Define and detect polypharmacy with AI

#### Context

The aim of the AI axis is to define and detect polypharmacy associated with health outcomes. The definition of polypharmacy is expected to incorporate several medications, their type, dosage and duration of use, or specific combinations of medications. For example, based on the QICDSS database, the AI axis will discover medication combinations that are associated with an increased risk of death, hospitalizations or gradual deterioration of the patient state (e.g., worsening of the fragility index and other outcomes that are not binary, which will be identified by the Health axis, objective 2.1). The main challenge will be to identify clinically relevant correlations between the addition of the new medications and health events among the millions of observations, excluding multiple accidental correlations and coincidences that analysis of such a dataset will inevitably reveal.

#### Objectives and methodology

Objective 1.1 aims to define inappropriate polypharmacy using frequent pattern detection algorithms from big datasets. It involves discovering combinations of medications that will allow prediction of subsequent health events such as hospitalization or death. As benchmarks, we will evaluate algorithms such as Apriori [26], FP-Growth [27], and Pattern Fusion [28], which we will compare to the patterns discovered by more novel data mining algorithms based on genetic algorithms [29, 30] and reinforcement learning [31–33]. The obtained list of medication combinations will be refined by taking into consideration such attributes as the dosage of each medication, the duration of treatment, and the order in which the medications were prescribed, and all that in the context of up to 20 years of follow-up observations. The collaboration between the AI axis and the Health axis will be necessary to determine which medications and treatment attributes should be prioritized.

Objective 1.2 will explore the social dimension of the data. Some subgroups of the population may be more sensitive to specific medication combinations and their outcomes, for example based on their age or medical conditions. The challenge will consist in finding an appropriate representation of individuals allowing them to be partitioned depending on demographic or geographic characteristics. This entails integrating heterogeneous individual-level data that contain attributes that are numeric (e.g., postal code that can be used to derive Euclidean distance between two individuals), binary (e.g., sex), or that describe temporal dimension (e.g., time when a medication was taken and duration of its use). Deep neural networks have previously been proven to learn patient representation from electronic health records and will be considered for this task of clustering [34]. The frequent pattern detection algorithms developed in objective 1.1 will be applied independently to each subpopulation retrieved. They will help to determine which polypharmacy problems are present in these specific subpopulations and their differences from the general population studied in objective 1.1. We will also investigate the possibility of predicting the effect of treatment over time, considering the individual characteristics of a patient. Specifically, we will use recurrent neural networks that have been effective in predicting the series of events from electronic health records [35, 36]. Potential biases that can arise both from the composition of the data and their temporal nature will be of particular interest. For example, the older population in Quebec is mainly white and well-off. Therefore, the neural network is at risk of learning from decisions that benefit this group to the detriment of others. Close collaboration with the Health axis will make it possible to decide which attributes should be prioritized at the beginning of the research, and collaboration with the Law&Ethics axis will prevent the inclusion of an accidental discriminatory bias.

Objective 1.3 will focus on the challenges related to the use of AI strategies in the healthcare, more specifically on the problem of explainability. While demonstrating better predictive performance, the results obtained with neural networks are difficult to explain, which is a limiting factor when one needs to justify why a treatment is being recommended to a patient. Moreover, the complexity of the patterns discovered in the previous objectives and the potential presence of confounding factors [37] can make them very difficult for users to interpret [38]. This is what makes these systems “black boxes”; they give highly predictive solutions, but it is difficult or impossible for the users to understand how they arrive at them, which is a clear problem when patients are asked to trust their health and potentially their lives to these solutions. We will explore different strategies to make the models more explainable and interpretable. The system will notably be characterized in terms of explicitness, faithfulness, and stability [39].

*Data preprocessing*: The data are currently in the form of relational database tables that contain institutional codes. It will be necessary to transform them into groups of items with different levels of granularity of detail. The institutional codes will be arranged in a hierarchical or in ontological manner to allow algorithms to find similar health conditions and medications with the same mechanisms of action or active ingredients. The data preprocessing is a prerequisite for the proper functioning and success of the algorithms of the three objectives. This work will be carried out in accordance with FAIR principles (Findable, Accessible, Interoperable, Reusable)[40] and international standards (SNOMED CT, LOINC, FHIR) [41, 42]. By documenting the actions and activities carried out, we will promote continuous data valuation, reuse, and development.

### Health axis: Development of polypharmacy indicators

#### Context

The Health axis aims to develop polypharmacy indicators that will be useful for population-level surveillance in order to guide health promotion and disease prevention interventions, and to support clinical practice. Thus, the Health axis will be responsible for translating the information generated by the AI axis into relevant and understandable indicators that will be socially acceptable through recommendations of the Law&Ethics axis (Fig. 1).

#### Objectives and methodology

Objective 2.1 will define health outcomes that reflect a continuum of changes in health status. Since deaths, and to a lesser extent, hospitalizations, are relatively rare events, only a small portion of the population will be affected. We will identify several types of metrics, such as frailty indices or composite health outcomes (e.g., frequency of medical and emergency room visits due to decompensation of a health condition) that will be useful to define various intermediate stages between states of good health, illness, and death. This approach will enable evaluation of the health outcomes for all individuals. In addition, we will consider institutionalization in the long-term care facilities as one of potentially “negative” health outcomes. The defined continuum of health outcomes will be transmitted to the AI axis and will be used to validate previous algorithms or create new ones, in order to identify polypharmacy patterns and characteristics associated with said outcomes.

Objective 2.2 will interpret the frequent patterns and deep representations discovered in the AI axis and transform them into polypharmacy indicators. The Law&Ethics axis will ensure that these indicators are socially acceptable, fair, and do not ignore potential harmful impacts on small subpopulations. Thereafter, each indicator will be validated for clinical and organizational utility on a test set: the data collected between 2016 and 2019. Predictive models will measure the indicator’s effectiveness to predict health events, such as mortality and hospitalizations (multivariable Cox model), and continuum of health outcomes (regression analysis).

Cox models will use age as a time scale [43, 44]; all the models will be adjusted for the initial material and social deprivation indices [24], the initial comorbidity index [25] and any other variable deemed relevant by the collaborating experts. Particular attention will be paid to the effect of sex, assessed with stratified analysis. The indicators will be compared according to their discriminating power, i.e., the ability of the model to adequately distinguish between subjects with and without the specific health outcome, using the C-index and the Nagelkerke R^2^, their calibration (slope and calibration curve), and their overall predictive power (observed probabilities compared to predicted probabilities) [45]. Statistics corrected for overfitting will be obtained using bootstrap [46]. Finally, we will evaluate the temporal stability of polypharmacy indicators by comparing their predictive capacities for cohorts from previous years (e.g., 2005–2008, 2010–2013).

An explanatory model will guide the estimation of causal links between indicators and health events. Given the variable nature of medication exposure and potentially confounding covariates, such as comorbidities, the relationship between polypharmacy indicators and clinical outcomes could be subject to time-dependent confounding [47]. We will use causal inference methods such as marginal structural models to estimate the effect of our polypharmacy indicators on hospitalizations, deaths and other health outcomes identified in objective 2.1 [48]. The fraction of events attributable to inappropriate polypharmacy, as defined by each polypharmacy indicator, will be calculated, making it possible to quantify the impact in terms of public health [49].

Finally, the robustness of the results to unmeasured confounding factors will be assessed using the E-value [50, 51]. The E-value indicates the strength of association between an unmeasured confounding variable and (1) the exposure and (2) the event that would be necessary to cancel the association between the exposure and the event.

The indicators will be used for the surveillance that is carried out by the INSPQ. These indicators should be useful and understandable for public health workers, decision-makers, clinicians, and the patients. Thus, the choice of indicators will be made jointly with these potential users with the help of consensus procedures, notably the RAND/Delphi approach [52, 53]. We will recruit approximately twenty participants: patients, clinicians (family doctors, geriatricians, pharmacists, nurses), researchers, public health decision-makers and surveillance specialists to evaluate the proposed indicators. We will assess the basic qualities of the suggested indicators, including validity and reliability, ease of use and ease of understanding [54, 55].

### Law&Ethics axis: Law, ethics, and social acceptability

#### Context

The collection, use, and communication of personal health information to train AI algorithms raise unique legal and ethical concerns and require a responsible approach [56]. Therefore, the Law&Ethics axis of the research project is necessary to ensure the legal conformity of the research process, to validate the usefulness of the results, and the acceptability and legitimacy of their application. This axis will involve researchers, practitioners, and patients.

#### Objectives and methodology

Objective 3.1 will aim to verify that the use of new correlations or additional emerging attributes in the AI Axis is carried out in an ethical manner. Particular attention will be paid to a set of risks associated with stigmatization, re-identification, and fairness. Stigmatization occurs when data reinforces the labelling of population subgroups, that are often already disadvantaged or taken responsible for their health problems. Data linkage operations may increase the risk of identifying patients from data sets that were previously anonymized. It may happen when few of them share specific characteristics, for example, due to the frequency of the studied phenomena, their nature, or the size of the territory where these phenomena are observed. Finally, the issue of fairness can arise when certain population subgroups are unduly excluded from analysis or under-represented in the dataset. The Law&Ethics axis will act as an internal ethics committee for the project, allowing researchers involved in the AI axis to handle these risky situations.

Objective 3.2 will consist of the ethical and social validation of the indicators developed in the Health axis. The approach will be based on grids developed by the INSPQ [57, 58]. Two data collection methods are planned: semi-structured interviews and focus groups. The interviews will be conducted with 30 people. There will be 5 focus groups of 4 to 6 participants. Using these results, we will build a specific ethic assessment grid that will allow weighting individual and collective interests related to the use of polypharmacy indicators and assessing social and ethical validity in the context of surveillance.

Objective 3.3 will focus on the development of markers and guidelines that could frame the ethical reasoning in the context of polypharmacy and AI. We will adopt an approach inspired by narrative ethics, a method primarily developed in medicine, which allows researchers and patients to narrate the challenges and ethical questions they were confronted with during a particular project [59]. This method is mainly used to investigate emerging and unique ethical issues. The discussions of the experiences lived by researchers and other participants will shed light on many nuances and ethical subtleties of this complex and new practical case [60, 61]. The publication of the ethical considerations will provide the opportunity to highlight certain pitfalls to be avoided or certain attitudes to be adopted.

### Patient and public involvement

Patients will be involved in the validation process of polypharmacy indicators and in discussions within the Law&Ethics axis. Among other things, they will ensure that the developed indicators meet their priorities and preferences.

## Discussion

The impact of use of various combinations of multiple medications on individual health and health care system is largely unknown. This research program will be performed by a multi-disciplinary team consisting of specialists in AI, health data, statistics, pharmacy, public health, law, and ethics. Such diversity of expertise will allow investigation of polypharmacy from different points of view and will contribute to a deeper understanding of the clinical, social, and ethical issues surrounding polypharmacy and its surveillance, as well as the use of AI for health record data.

In addition, the polypharmacy indicators will be developed in partnership with patients, clinicians, researchers, and public health decision-makers, ensuring the proposed indicators to be valid, reliable, understandable, and simple for their use in surveillance and clinics.

One of the strengths of the research program is that it will be conducted on an extensive database that contains time-stamped medical and sociodemographic information covering more than 90% of older adults in the province of Quebec, Canada, over two decades.

The main limitation of the research is that the data do not contain variables related to race, ethnicity, gender, health habits, and specific clinical data that could influence both medication use and polypharmacy, and their associated outcomes.

### Anticipated challenges and mitigation strategies

Each of the axes will meet challenges specific to their objectives. The complexity of the data and their interpretation will make it necessary to test different methodological approaches and will involve a lot of feedback between the axes. The older population is heterogeneous in terms of physiological, psychological, social, and cultural peculiarities. It will not be possible to directly investigate the role of individual factors such as life habits and genetics because they are not contained in the databases. Thus, since many factors other than medications may influence the health outcomes, the interpretation will have to be made with caution.

Similarly, the data mining process may generate a very large number of medication use patterns. Among those latter, some may be ambiguous, inconsistent, or even absurd. Therefore, the interpretation of the results will be of particular importance and will be jointly conducted by researchers and clinicians that will use their technical expertise and clinical experience.

Our team is committed to popularizing the procedures carried out so that all stakeholders can understand the processing that was done with the data. We are also aware that the advent of new techniques can open the path towards inconsistent approaches, and this is the reason why the Law&Ethics team will produce new knowledge, markers, and guidelines for future research in this area.

Our results will be published in peer reviewed scientific journals, presented at local and international scientific meetings, and shared with clinicians and healthcare professionals via the INSPQ leaflets, website, and public health information center.

## Data Availability

Data from the QICDSS is not available publicly.

## Abbreviations

AI: Artificial intelligence
INSPQ: National Public Health Institute of Quebec
QICDSS: Quebec Integrated Chronic Disease Surveillance System
RAMQ: Régie de l’assurance maladie du Québec
MSSS: Ministry of Health and Social Services

## References

[1] Canadian Institute for Health Information. Prescribed drug spending in Canada, 2020: A focus on public drug programs. Ottawa, ON: CIHI; 2020.

[2] OECD. Pharmaceutical spending (indicator); 2019. Available from: https://data.oecd.org/healthres/pharmaceutical-spending.htm. Accessed 17 February 2021.

[3] Schumock GT, Stubbings J, Hoffman JM, Wiest MD, Suda KJ, Rim MH (2019). National trends in prescription drug expenditures and projections for 2019. Am J Health Syst Pharm.

[4] Kesselheim AS, Avorn J, Sarpatwari A (2016). The high cost of prescription drugs in the United States: origins and prospects for reform. JAMA.

[5] Linnér L, Eriksson I, Persson M, Wettermark B (2020). Forecasting drug utilization and expenditure: ten years of experience in Stockholm. BMC Health Serv Res.

[6] Jo J, Kim Y, Paek K, Bea M, Chun K, Lee S (2016). Factors contributing to increases in prescription drug expenditures borne by National Health Insurance in South Korea. Yonsei Med J.

[7] Hovstadius B, Petersson G (2013). The impact of increasing polypharmacy on prescribed drug expenditure-a register-based study in Sweden 2005–2009. Health Policy.

[8] Canadian Institute for Health Information. Drug use among seniors in Canada, 2016. Ottawa, ON: CIHI; 2018.

[9] Khezrian M, McNeil CJ, Murray AD, Myint PK (2020). An overview of prevalence, determinants and health outcomes of polypharmacy. Ther Adv Drug Saf.

[10] Davies LE, Spiers G, Kingston A, Todd A, Adamson J, Hanratty B (2020). Adverse outcomes of polypharmacy in older people: systematic review of reviews. J Am Med Dir Assoc.

[11] Mohamed MR, Ramsdale E, Loh KP, Arastu A, Xu H, Obrecht S (2020). Associations of polypharmacy and inappropriate medications with adverse outcomes in older adults with cancer: a systematic review and meta-analysis. Oncologist.

[12] Saastamoinen LK, Verho J (2015). Register-based indicators for potentially inappropriate medication in high-cost patients with excessive polypharmacy. Pharmacoepidemiol Drug Saf.

[13] Hanley G, Morgan S (2009). Chronic catastrophes: exploring the concentration and sustained nature of ambulatory prescription drug expenditures in the population of British Columbia. Can Soc Sci Med.

[14] Rankin A, Cadogan CA, Patterson SM, Kerse N, Cardwell CR, Bradley MC, et al. Interventions to improve the appropriate use of polypharmacy for older people. Cochrane Database Syst Rev. 2018 Sep 3;9(9):CD008165. doi: 10.1002/14651858.CD008165.pub4. 10.1002/14651858.CD008165.pub4 PMC6513645 30175841

[15] Sirois C, Domingues NS, Laroche M-L, Zongo A, Lunghi C, Guénette L (2019). Polypharmacy definitions for multimorbid older adults need stronger foundations to guide research, clinical practice and public health. Pharmacy.

[16] Masnoon N, Shakib S, Kalisch-Ellett L, Caughey GE (2017). What is polypharmacy? A systematic review of definitions. BMC Geriatr.

[17] Sirois C, Simard M, Gosselin E, Gagnon M-E, Roux B, Laroche M-L (2019). Mixed bag "Polypharmacy": Methodological pitfalls/challenges of this exposure definition. Curr Epidemiol Rep.

[18] Cadogan C, Ryan C, Hughes C (2016). Appropriate polypharmacy and medicine safety: when many is not too many. Drug Saf.

[19] Kouladjian L, Hilmer S, Chen T, Le Couteur DG, Gnjidic D (2014). Assessing the harms of polypharmacy requires careful interpretation and consistent definitions. Br J Clin Pharmacol.

[20] Sirois C, Lunghi C, Laroche ML, Maheux A, Frini A (2019). The delicate choice of optimal basic therapy for multimorbid older adults: a cross-sectional survey. Res Soc Adm Pharm.

[21] Thacker S, Berkelman R. History of public health surveillance. In: Halperin W, Baker EL, editors. Public Health Surveillance. New York: Van Norstrand Reinhold; 1992.

[22] Centre for Disease Control and Prevention. Updated guidelines for evaluating public health surveillance systems. Recommendations from the Guidelines Working Group. MMWR Recommendations and Reports. 2001;50(RR13):1–35. 18634202

[23] Blais C, Jean S, Sirois C, Rochette L, Plante C, Larocque I (2014). Quebec integrated chronic disease surveillance system (QICDSS), an innovative approach. Chronic Dis Inj Can.

[24] Pampalon R, Hamel D, Gamache P, Raymond G (2009). A deprivation index for health planning in Canada. Chronic Dis Can.

[25] Simard M, Sirois C, Candas B (2018). Validation of the combined comorbidity index of Charlson and Elixhauser to predict 30-day mortality across ICD-9 and ICD-10. Med Care.

[26] Agrawal R, Srikant R. Fast algorithms for mining association rules. Proceedings of the 20th international conference of very large data bases. 1994;1215:487–99.

[27] Borgelt C. An implementation of the FP-growth algorithm. Proceedings of the 1st international workshop on open-source data mining: frequent pattern mining implementations, ACM. 2005:1–5.

[28] Zhu F, Yan X, Han J, Yu PS, Cheng H, editors. Mining colossal frequent patterns by core pattern fusion. 2007 IEEE 23rd international conference on data engineering; 2007 15–20 April 2007.

[29] Mitchell M (1996). An introduction to genetic algorithms.

[30] Falkenauer E (1997). Genetic algorithms and grouping problems.

[31] Chen S, Lin T, King I, Lyu MR, Chen W. Combinatorial pure exploration of multi-armed bandits. In Proceedings of NeurIPS. 2014

[32] Kaufmann E, Koolen W. Monte-Carlo Tree Search by Best Arm Identification. In Proceedings of NeurIPS. 2017

[33] Kallus N, Zhou A. Confounding-Robust Policy Evaluation in Infinite-Horizon Reinforcement Learning. In Proceedings of NeurIPS. 2020

[34] Miotto R (2016). Deep patient: AN unsupervised representation to predict the future of patients from the electronic health records. Sci Rep.

[35] Hermans M, Schrauwen B (2013). Training and analysing deep recurrent neural networks. Adv Neural Inf Process Syst.

[36] Choi E, Schuetz A, Sun J (2016). Doctor AI: predicting clinical events via recurrent neural networks. Proc Mach Learn Healthc JMLR W&C.

[37] D'Amour A. On multi-cause causal inference with unobserved confounding: counterexamples impossibility, and alternatives. Proceedings of the 22nd international conference on artificial intelligence and statistics (AISTATS) 2019, Naha, Okinawa, Japan PMLR. 2019;89.

[38] Topol E (2019). High-performance medicine: the convergence of human and artificial intelligence. Nat Med.

[39] Alvarez-Melis D, Jaakkola T. Towards robust interpretability with self-explaining neural networks. Proceedings of the 32nd conference on neural information processing systems (NeurIPS 2018), Montréal, Canada. 2018.

[40] Wilkinson MD, Dumontier M, Aalbersberg IJ, Appleton G, Axton M, Baak A (2016). The FAIR guiding principles for scientific data management and stewardship. Scientific Data.

[41] Bodenreider O, Cornet R, Vreeman DJ (2018). Recent developments in clinical terminologies: SNOMED CT, LOINC, and RxNorm. Yearb Med Inform.

[42] Lehne M, Luijten S, Imbusch VFG, P, Thun S,  (2019). The Use of FHIR in digital health: a review of the scientific literature. Stud Health Technol Inform.

[43] Thiébaut A, Bénichou J (2004). Choice of time-scale in Cox’s model analysis of epidemiologic cohort data: a simulation study. Stat Med.

[44] Kom E, Graubard B, Midhyne D (1997). Time-to-event analysis of longitudinal follow-up of a survey: choice of the time-scale. Am J Epidemiol.

[45] Moore L, Lauzier F, Stelfox H, Kortbeek J, Simons R, Berthelot S (2016). Derivation and validation of a quality indicator to benchmark in-hospital complications among injury admissions. JAMA Surg.

[46] Harrell FJ. Regression modeling strategies: with applications to linear models, logistic and ordinal regression, and survival analysis. Springer N-Y, editor; 2015.

[47] Mansournia MA, Etminan M, Danaei G, Kaufman JS, Collins G (2017). Handling time varying confounding in observational research. BMJ.

[48] Xiao Y, Abrahamowicz M, Moodie EE (2010). Accuracy of conventional and marginal structural Cox model estimators: a simulation study. Int J Biostat.

[49] Sjölander A, Vansteelandt S (2017). Doubly robust estimation of attributable fractions in survival analysis. Stat Methods Med Res.

[50] VanderWeele TJ, Ding P (2017). Sensitivity analysis in observational research: introducing the E-Value. Ann Intern Med.

[51] Haneuse S, VanderWeele TJ, Arterburn D (2019). Using the E-Value to assess the potential effect of unmeasured confounding in observational studies. JAMA.

[52] Fink A, Kosecoff J, Chassin M, Brook R (1984). Consensus methods: characteristics and guidelines for use. Am J Public Health.

[53] Fitch K, Bernstein S, Aguilar M, et al. The RAND/UCLA appropriateness method user's manual. Santa Monica, CA: RAND; 2001.

[54] Parrish R (2010). Measuring population health outcomes. Prev Chronic Dis.

[55] Betancourt M, Roberts K, Bennett T, Discoll E, Jayaraman G, Pelletier L (2014). Surveillance des maladies chroniques au Canada: Cadre conceptuel d'indicateurs des maladies chroniques. Maladies chroniques au Canada.

[56] Price II WN. Black-Box Medicine. Harv. J. L. & Tech.; 2015. pp. 419–67.

[57] Institut national de santé publique du Québec. Dimension éthique de la stigmatisation en santé publique. Outil d’aide à la réflexion. Gouvernement du Québec.

[58] Comité d'éthique de santé publique. Guide sur l’élaboration de plans de surveillance, incluant les notions éthiques à considérer, et les modalités de dépôt au Comité d’éthique de santé publique (CESP). Gouvernement du Québec, 2017.

[59] La NC (2015). philosophie dans l’éthique narrative. Narration, prise de décision et souffrance morale. Ethics Med Public Health..

[60] Le Sommer-Péré M, Gagnon J, Stiegler B (2017). Réflexion interdisciplinaire et interculturelle sur l’éthique du soin. L’expérience d’échanges France-Québec (2009–2015). Éthique Santé..

[61] Langlois L, Tanguay D, Fillion L, Robitaille M (2015). La sensibilité éthique. Une fenêtre pour combattre les inégalités de pouvoir entre les groupes. Recherches féministes..

